# Successful percutaneous nephrolithotomy in patient with ipsilateral dual kidney transplant, a case report

**DOI:** 10.1016/j.eucr.2024.102812

**Published:** 2024-07-22

**Authors:** Thomas Cato, William Crasto, Carly Ulrich, Ryan Fitzwater

**Affiliations:** a Charleston Area Medical Center Department of Urology, United States; b William Carey University College of Osteopathic Medicine, United States

**Keywords:** Percutaneous nephrolithotomy, Kidney, Allograft, Stone, Transplant

## Abstract

Percutaneous Nephrolithotomy is a minimally-invasive procedure used in the setting of complex stone burden. Among its uses, PCNL can be employed to treated renal allograft calculi. This case presented a unique challenge and a rare usage of PCNL that involved removal of a 2.6 cm stone that presented in a 43-year-old male with dual renal allografts. The unique location of the allograft presented challenges that were navigated successfully with an uneventful postoperative course and no residual stone burden. The utilization of PCNL to treat calculi in dual renal allografts has been minimally reported in the literature.

## 1 Introduction

Percutaneous Nephrolithotomy (PCNL) is a procedure that utilizes a minimally invasive approach in the management of complex kidney stones. Its application in renal calculi, specifically in the setting of donor renal allografts, has presented unique considerations and challenges. Dual kidney transplantation (DKT), which involves the transplantation of two allograft kidneys into a single recipient, has emerged as a valuable method in the treatment of end-stage renal disease (ESRD). In addition to increasing nephron mass, DKT has superior graft viability when compared to single kidney transplantation in long-term analysis.^1^ Furthermore, DKT can reduce the waiting time for patients needing a kidney transplant. When compared to single kidney transplantation, DKT is susceptible to the same challenges and complications, including transplant lithiasis. Procedures used for stone burden in native kidneys can typically be applied to renal allografts, including pyelolithotomy, ureteroscopy, extracorporeal shockwave lithotripsy (ESWL), and percutaneous nephrolithotomy (PCNL).^2^ When compared to other techniques, PCNL has illustrated a statistically significant stone-free rate (SFR) at three months.^3^ In addition to its minimally invasive nature, PCNL offers an approach that can manage a large stone burden in transplant kidneys and navigate the anatomical abnormalities of this patient subset. This case represents the utilization of PCNL for the treatment of urolithiasis in dual transplant kidneys, a unique challenge that is minimally reported in the literature.

## 2 Case summary

**Fig. 1:**
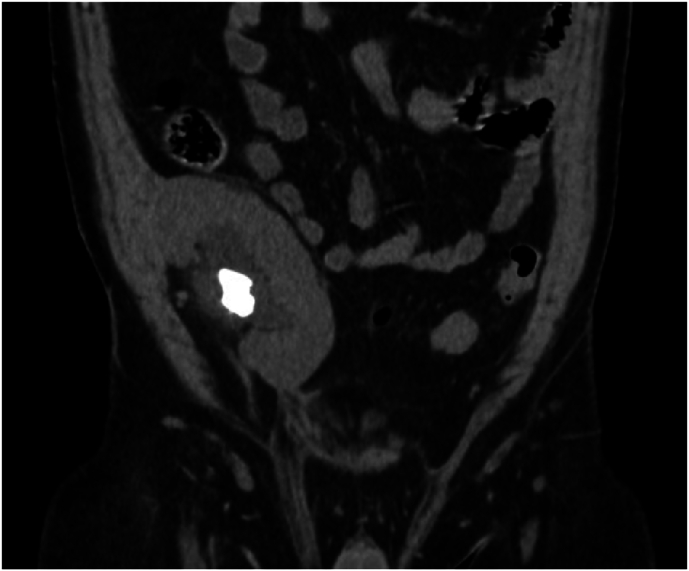
– CT revealing a 2.6 cm staghorn calculi found in the transplanted kidney.

**Fig. 2:**
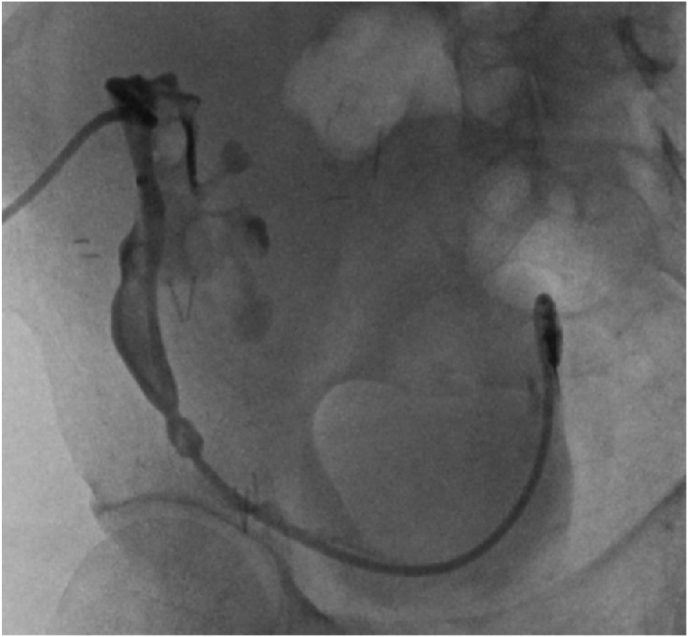
– Placement of the nephroureteral stent by Interventional Radiology.

This patient is a 43-year-old male with a history of end-stage renal disease who underwent dual deceased donor kidney transplant in June 2023 with both kidneys being placed into the right iliac fossa. To allow for adequate space for placement of both kidneys, the peritoneum was incised intraoperatively by the kidney transplant surgeon. Following surgery, he was found to have a large staghorn stone in his superomedially located transplant kidney within the right iliac fossa (Fig. 1). Plans were made for percutaneous nephrolithotomy for treatment due to large stone burden. An initial attempt was made to gain access *via* needle placement under direct visualization with ultrasound, but this was unsuccessful due to overlying bowel from incision of peritoneum at time of kidney transplantation. Interventional Radiology was then consulted to aid access of this transplant kidney which was done successfully without complication or injury to overlying bowel. A nephroureteral stent was then placed successfully by Interventional Radiology (Fig. 2).

**Fig. 3:**
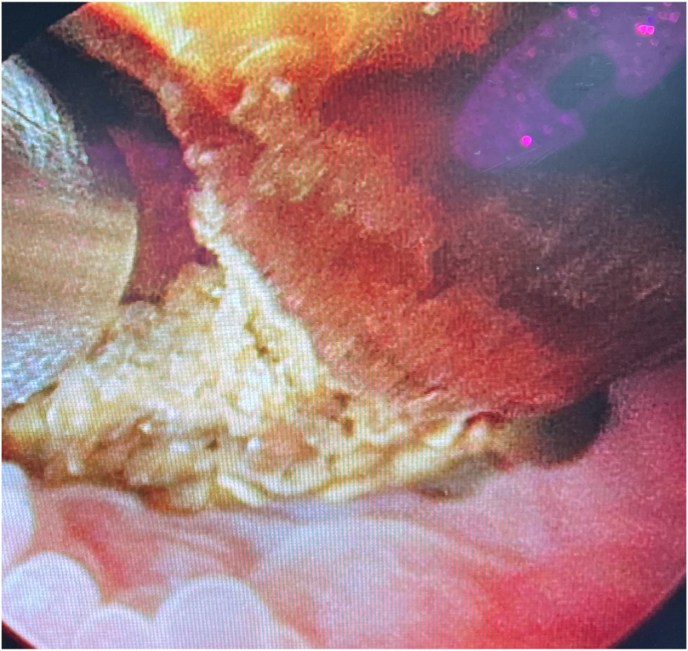
Pyeloscopy revealing a large staghorn stone within the renal pelvis.

Now that access was safely and adequately obtained, the patient was then taken to the PCNL room where he was placed on the table in the supine position. The nephroureteral stent was replaced with a wire and tract dilated to gain access to the upper pole of the transplant kidney for treatment of his large stone burden. The PCNL sheath was then advanced over balloon dilator to gain access and the nephroscope was subsequently inserted. Pyeloscopy revealed large staghorn stone within the renal pelvis approximately 2.6 cm in diameter (Fig. 3). A Lithoclast device was obtained and percutaneous nephrolithotomy was performed until all stone burden had been removed. A 5 French by 12 cm double-J ureteral stent was placed at the end of the case to allow for decompression of the collecting system, and patient's skin and subcutaneous tissues were closed in standard fashion. The patient progressed adequately after the procedure with no residual stone burden seen on renal ultrasound obtained at a two-month follow-up. Renal function remained at baseline with creatinine 1.0.

## 3 Conclusion

This case outlines a successful PCNL of a dual transplant kidney located within the same iliac fossa as the other transplant kidney. To our knowledge, this is the first case report detailing complex treatment of stone disease of this type. With the assistance of interventional radiology, safe and adequate access to transplant kidney is feasible in complex patients when traditional modalities for access fail. PCNL is a safe option for treatment of large stone burdens in patients with single or dual kidney transplants.

## CRediT authorship contribution statement

**Thomas Cato:** Writing – review & editing. **William Crasto:** Writing – review & editing, Writing – original draft. **Carly Ulrich:** Writing – review & editing. **Ryan Fitzwater:** Writing – review & editing.

## Declaration of competing interest

The authors declare that they have no known competing financial interests or personal relationships that could have appeared to influence the work reported in this article.

## References

[1] Khalil M.A.M., Tan J., Khan T.F.T., Khalil M.A.U., Azmat R. (2017). Dual kidney transplantation: a review of past and prospect for future. Int Sch Res Notices.

[2] Li X., Li B., Meng Y. (2020). Treatment of recurrent renal transplant lithiasis: analysis of our experience and review of the relevant literature. BMC Nephrol.

[3] Boissier R., Rodriguez-Faba O., Zakri R.H. (2023). Evaluation of the effectiveness of interventions on nephrolithiasis in transplanted kidney. Eur Urol Focus.

